# Doxorubicin Improves Cancer Cell Targeting by Filamentous Phage Gene Delivery Vectors

**DOI:** 10.3390/ijms21217867

**Published:** 2020-10-23

**Authors:** Effrosyni Tsafa, Kaoutar Bentayebi, Supachai Topanurak, Teerapong Yata, Justyna Przystal, Duriya Fongmoon, Nabil Hajji, Sajee Waramit, Keittisak Suwan, Amin Hajitou

**Affiliations:** 1 Phage Therapy Group, Department of Brain Sciences, Imperial College London, London W12 0NN, UK; md06673@uoi.gr (E.T.); kaoutar.bentayebi@gmail.com (K.B.); Teerapong.Y@chula.ac.th (T.Y.); justyna.przystal@googlemail.com (J.P.); s.waramit15@imperial.ac.uk (S.W.); 2 Department of Molecular Tropical Medicine and Genetics, Faculty of Tropical Medicine, Mahidol University, Bangkok 10400, Thailand; supachai.top@mahidol.ac.uk; 3 Department of Medical Services, Lampang Cancer Hospital, Ministry of Public Health, Lampang 52000, Thailand; duriya19@hotmail.com; 4 John Fulcher Neuro-Oncology Laboratory, Department of Brain Sciences, Imperial College London, London W12 0NN, UK; n.hajji@imperial.ac.uk

**Keywords:** doxorubicin, cancer, bacteriophage, targeted gene delivery

## Abstract

Merging targeted systemic gene delivery and systemic chemotherapy against cancer, chemovirotherapy, has the potential to improve chemotherapy and gene therapy treatments and overcome cancer resistance. We introduced a bacteriophage (phage) vector, named human adeno-associated virus (AAV)/phage or AAVP, for the systemic targeting of therapeutic genes to cancer. The vector was designed as a hybrid between a recombinant adeno-associated virus genome (rAAV) and a filamentous phage capsid. To achieve tumor targeting, we displayed on the phage capsid the double-cyclic CDCRGDCFC (RGD4C) ligand that binds the alpha-V/beta-3 (α_v_β_3_) integrin receptor. Here, we investigated a combination of doxorubicin chemotherapeutic drug and targeted gene delivery by the RGD4C/AAVP vector. Firstly, we showed that doxorubicin boosts transgene expression from the RGD4C/AAVP in two-dimensional (2D) cell cultures and three-dimensional (3D) tumor spheres established from human and murine cancer cells, while preserving selective gene delivery by RGD4C/AAVP. Next, we confirmed that doxorubicin does not increase vector attachment to cancer cells nor vector cell entry. In contrast, doxorubicin may alter the intracellular trafficking of the vector by facilitating nuclear accumulation of the RGD4C/AAVP genome through destabilization of the nuclear membrane. Finally, a combination of doxorubicin and RGD4C/AAVP-targeted suicide gene therapy exerts a synergistic effect to destroy human and murine tumor cells in 2D and 3D tumor sphere settings.

## 1. Introduction

Gene therapy has been attempted against cancer for the past 28 years, with more than 67% of clinical trials of gene therapy designed to treat cancer patients [1]. However, progress has been hindered, mostly by a lack of tumor-selective gene delivery vectors efficient via clinical systemic routes and by problems with repeated vector administrations [2,3]. Currently, the most common vectors are eukaryotic viruses, because they can enter cells and deliver genes as part of the natural infection process. Contrarily, we developed a unique prokaryotic viral-based vector of targeted intravenous gene delivery to solid tumors by using the safe and harmless nonpathogenic filamentous fd phage, which lacks native tropism to human tissues [4]. The vector is a hybrid [5,6] in which the phage capsid incorporates hybrid genomes of single-stranded human adeno-associated virus (AAV-2) DNA and the fd phage single-stranded genome. Moreover, to allow entry into cancer cells, we used the Nobel Prize-awarded phage display technology to engineer a phage capsid displaying the double-cyclic RGD4C ligand that binds the α_v_β_3_ integrin cell surface receptor overexpressed on tumor cells and tumor vasculature in most cancers but is barely detectable in healthy tissues [7,8,9]. Upon cell entry of the hybrid RGD4C/AAV-phage (RGD4C/AAVP), the AAV transgene cassette is released, resulting in gene expression in tumors from a cytomegalovirus, *CMV*, promoter [5,6]. We have reported that this vector targets numerous cancer models in vivo upon intravenous delivery [6,7,8,9,10,11,12,13,14,15,16,17,18,19]. A study in pet dogs with spontaneous cancers proved the safety of repeated vector dosing, resulting in complete tumor eradication in a few dogs with aggressive cancers [20]. Phage vectors have several advantages over existing delivery systems, such as the production at high titers and a historic safety profile (being safely administered to humans over years to treat bacterial infections); they are cost-effective and have no native tropism for human tissues, allowing their systemic delivery. Additionally, we have reported that repeated administrations of RGD4C/AAVP showed antitumor efficacy in immunocompetent mice and domesticated dogs, despite Immunoglobulins G (IgGs) against the phage capsid [5,20]. However, the phage has evolved to infect bacteria only, with no optimized strategies to deliver genes to mammalian cells. We have investigated various steps of gene delivery upon RGD4C/AAVP vector treatment of cells and found that the main extracellular limiting steps are vector diffusion through the extracellular matrix (ECM) and binding to the cell surface receptors caused by repulsion between the negatively charged phage capsid and mammalian cell surface membranes [21,22]. Following internalization, we reported the endosome/lysosome degradative pathway and proteasome degradation as major intracellular barriers to gene delivery by the vector [23,24]. To date, we have further refined the RGD4C/AAVP delivery technology and combined the vector with drug treatment, such as collagenase and hyaluronidase enzymes for increased diffusion through the ECM, cationic polymers to alter the phage electrostatic charge, chloroquine for endosomal escape, proteasome blocking agents and histone deacetylase inhibitors [21,22,23,24,25]. All these studies showed dramatic enhancement of gene transfers. Furthermore, we recently reported multifunctional RGD4C/AAVP vectors combining a multiple fusion peptide display on various coated proteins, which confer the ability of RGD4C/AAVP to escape from the endosome-lysosomal degradative pathway, alter the capsid charge and increase the vector stability [9]. Moreover, to enhance therapeutic gene transcription from the vector in the nucleus, we replaced the *CMV* promoter with a tumor-activated and chemotherapy-induced promoter of the glucose-regulated protein *Grp78* [8,15]. This vector ensured further tumor selectivity, through transcriptional targeting, and provided a much longer lasting gene expression in tumors than the vector carrying a *CMV* promoter [15]. We showed that a low-dose temozolomide (TMZ) chemotherapy, used clinically to treat brain tumors, boosted gene expression from the RGD4C/AAVP-*Grp78* in human glioblastoma [8]. Repeated administrations of the TMZ-activated vector carrying the gene for the thymidine kinase of the Herpes Simplex Virus (*HSVtk*) resulted in glioblastoma eradication in combination with ganciclovir (GCV) [8]. In this present study, we sought to expand this approach and investigate a chemovirotherapy strategy that can be applied to a broad range of solid tumors. A combination of chemotherapeutic drugs with gene therapy viral vectors, chemovirotherapy, has been used as an approach to enhance cancer gene therapy and to reduce the dose of chemotherapy to a less toxic, as well as cost-effective, degree [8,26,27]. Thus, we combined RGD4C/AAVP with doxorubicin and tested the efficacy against tumor cells of human and murine origin. The cytostatic drug doxorubicin, one of the most effective neoplastic drugs, is a well-known chemotherapeutic agent used in the treatment of a wide variety of cancers for over 30 years, mainly in combination with other drugs against solid tumors [28,29]. Indeed, while it provides a cure in some cases, doxorubicin is very toxic on noncancerous cells, forcing dose-limiting treatments and combinations with other anticancer agents [29]. We found that doxorubicin improved gene delivery by RGD4C/AAVP without affecting the vector selectivity for cancer cells, subsequently enhancing tumor cell killing. We also investigated the effects on vector cellular trafficking, since doxorubicin can alter the intracellular trafficking of eukaryotic viral vectors [30], and uncovered that destabilization of the nuclear envelope is one mechanism by which doxorubicin might enhance gene delivery.

## 2. Results

### 2.1. Doxorubicin Increases Targeted Gene Transfer by RGD4C/AAVP in Rat Glioma (9L) and Human Melanoma (M21) Cancer Cells In Vitro

**Figure 1:**
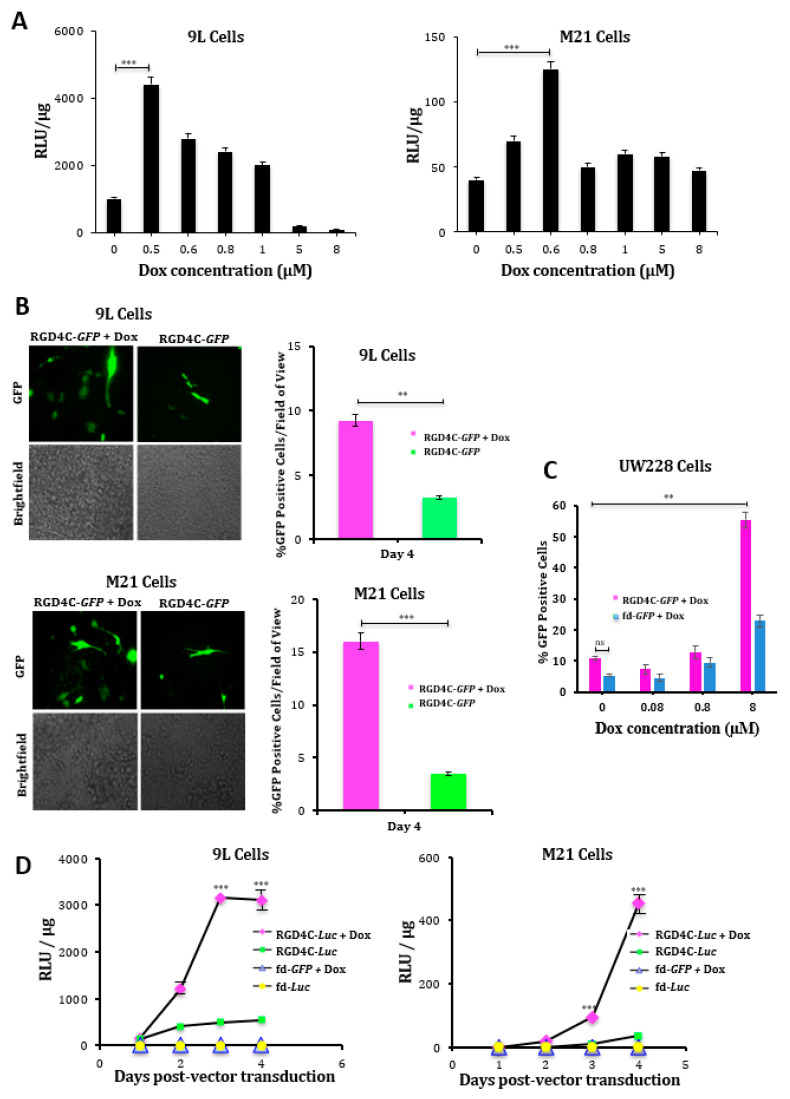
Doxorubicin (Dox) boosts targeted gene delivery by the RGD4C/ adeno-associated virus phage (AAVP) vector in rat glioma (9L) and human melanoma (M21) cancer cells. (**A**) Determination of optimal Dox doses in 9L and M21 cells using a luciferase assay. The 9L and M21 cells were transduced with the targeted RGD4C/AAVP-*Luc* (*luciferase*) vector carrying a luciferase (*Luc*) reporter gene, in combination with increasing concentrations of Dox ranging from 0.5 to 8 μM for 24 h. To evaluate gene delivery, a luciferase assay was performed at day 3 post-transduction. The results were normalized to the amount of protein, as determined by the Bradford assay, and shown as relative luminescence units (RLU)/μg of protein. (**B**) Evaluation of the gene expression in 9L and M21 cells in the presence of optimal Dox concentrations (0.5 μΜ for 9L and 0.6 μΜ for M21 cells). Tumor cells were plated in 48-well plates and transduced with the targeted RGD4C/AAVP-*green fluorescent protein* (*GFP*) (RGD4C-*GFP*) vector carrying a *GFP* reporter gene in the presence or absence of Dox. GFP expression was evaluated by fluorescent microscopy at day 4 post-vector transduction. GFP expression is also displayed as the average of GFP-positive cells in five fields of view of treated 9L and M21 cells. Original magnification ×200 for GFP and ×40 for brightfield images. (**C**) Fluorescence-activated cell sorting (FACS) analysis of GFP expression in UW228 medulloblastoma cells at day 5 post-transduction with RGD4C-*GFP* or nontargeted (fd-*GFP*) vectors with varying concentrations of Dox ranging from 0.08 to 8 μM for 24 h. (**D**) Evaluation of the effect of Dox on gene delivery over a time course. The 9L and M21 cells were plated in 48-well plates and transduced with the targeted RGD4C/AAVP-*Luc* (RGD4C-*Luc*) vector or control nontargeted AAVP-*Luc* (fd-*Luc*) in serum-free medium in the presence or absence of Dox 0.5 μΜ and 0.6 μΜ for 9L and M21 cells, respectively. Luciferase measurement assays were performed at various time points from days 1 to 4 post-vector transduction and normalized to protein concentrations. Results are shown as RLU per 1 μg of protein. All experiments were repeated twice, in triplicates, and the results shown are representative of one experiment. ** *p* < 0.01 and *** *p* < 0.001.

First, we determined the concentrations of doxorubicin that give the optimal gene delivery efficiency by RGD4C/AAVP in 9L and M21 tumor cells. Cells were transduced with vector carrying the firefly *luciferase*, *Luc*, reporter gene (RGD4C/AAVP-*Luc*) in combination with increasing concentrations of doxorubicin ranging from 0.5 to 8 μM for 24 h. At day three post-vector transduction, we found that 0.5 μM for 9L and 0.6 μΜ for M21 cells were the optimal doxorubicin concentrations that generated the highest *Luc* expression by the vector (Figure 1A). Next, we used these optimal doxorubicin doses and carried out a broader investigation of the effect of doxorubicin on gene delivery by RGD4C/AAVP by using two different reporter genes performing time course gene delivery experiments and testing the efficacy both in 9L and M21 tumor cells. First, we used vectors expressing a reporter gene of the *green fluorescent protein* (RGD4C/AAVP-*GFP*). At day four post-vector transduction, we observed more GFP expression in 9L and M21 tumor cells treated with a combination of RGD4C/AAVP-*GFP* and doxorubicin than in cells transduced with the RGD4C/AAVP-*GFP* vector alone (Figure 1B). To further evaluate doxorubicin effects on gene delivery levels, we repeated the GFP reporter gene expression experiments in the presence of increasing doxorubicin concentrations on the UW228, as an in vitro model of human medulloblastoma, which is the most common brain cancer in children. Herein, we used a fluorescence-activated cell sorting (FACS) analysis of GFP expression in UW228 cells and showed a substantial increase of GFP-expressing cells by doxorubicin, reaching 55% GFP-positive UW228 cells in the presence of 8-μM doxorubicin as compared to 11% of GFP-expressing cells treated with the vector alone (Figure 1C). Next, we used the RGD4C/AAVP-*Luc* vector and monitored luciferase expression over a time course (Figure 1D). Our data showed an increased luciferase expression by RGD4C/AAVP-*Luc* in 9L and M21 cells over time in the presence of doxorubicin as compared to treatment with the vector alone (Figure 1D). For example, at day four post-treatment, an analysis of *Luc* transgene expression showed that the combination treatment resulted in a ~5.3 and ~12-fold increase in *Luc* expression in 9L and M21 cells, respectively, compared to the RGD4C/AAVP-*Luc* vector alone. Moreover, in 9L cells, initiation of *Luc* expression occurred as early as day two post-vector transduction in the presence of doxorubicin (Figure 1D). Importantly, no *Luc* expression was detected in cells treated with the control nontargeted AAVP-*Luc* vector (lacking in RGD4C, fd-*Luc*) alone or in combination with doxorubicin, which shows that doxorubicin does not affect targeting of the RGD4C/AAVP vector and its selectivity for integrin-expressing cells.

### 2.2. Doxorubicin Drug Treatment Boosts Cancer Cell Death by AAVP-Mediated Suicide Gene Killing

**Figure 2:**
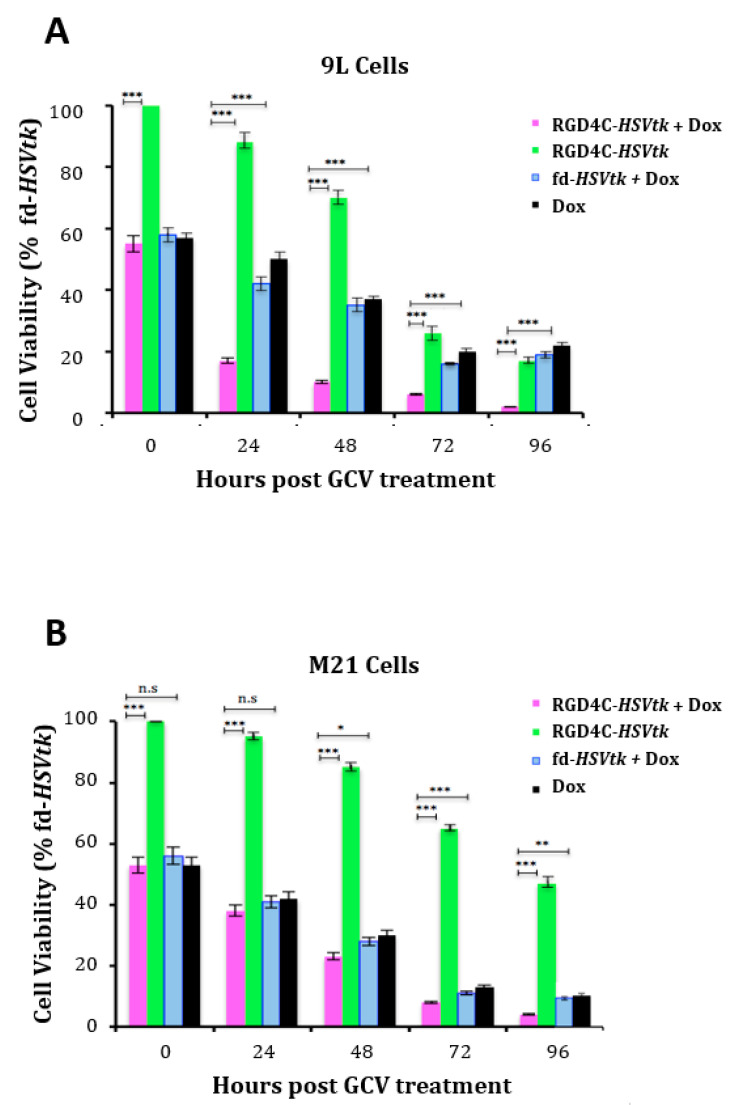
Dox amplifies the suicide gene killing of cancer cells by targeted RGD4C/AAVP-*HSVtk*. 9L (**A**) and M21 (**B**) cancer cells grown in 48-well plates, 60–80% confluent, were transduced with the targeted RGD4C/AAVP-*HSVtk* (RGD4C-*HSVtk*) vector carrying the *HSVtk* gene or control nontargeted AAVP-*HSVtk* (fd-*HSVtk*) in the presence or absence of Dox, 0.5 μΜ for 9L and 0.6 μΜ for M21 cells. The cells were treated with GCV (20 μM) at day 3 post-vector transduction and renewed daily. Cancer cell killing was quantified at 0, 24, 48, 72 and 96 h post-GCV treatment by using the trypan blue exclusion methodology. Results were normalized to the nontargeted fd-*HSVtk* vector. The experiments were repeated twice in triplicates, and the results shown are representative of one experiment. * *p* < 0.05, ** *p* < 0.01, *** *p* < 0.00 and n.s: nonsignificant.

We sought to investigate whether the enhanced vector-mediated gene delivery by doxorubicin is translated into an improved targeted killing of cancer cells by the vector carrying a therapeutic gene. We used the RGD4C*/AAVP-HSVtk* vector transferring the gene for *HSVtk* mutant SR39 [31], which kills cells in the presence of GCV. The HSVtk enzyme phosphorylates prodrug nucleoside analogs such as GCV and converts them into nucleoside analog triphosphates, which are then incorporated into the cellular genome, inhibit DNA polymerase and, subsequently, induce cell death by apoptosis [32]. Thus, 9L and M21 cells were treated with RGD4C/AAVP-*HSVtk* or control nontargeted vector fd-*HSVtk* in the presence or absence of doxorubicin. GCV (20 μM) was added after three days, and tumor cell death was evaluated at 0, 24, 48, 72 and 96 h following treatment with GCV. Data were normalized to the nontargeted fd-*HSVtk* vector that did not show any significant tumor cell death (Supplementary Figure S1). In both cancer cell lines, the combination treatment with doxorubicin and RGD4C/AAVP-*HSVtk* therapy resulted in greater cell killing compared to cells treated with RGD4C/AAVP-*HSVtk* or doxorubicin alone (Figure 2). For example, 92 h after GCV addition, we measured 99.7% and 96.5% cell death of 9L and M21 by the combination treatment, respectively, compared to 87% and 58.3% cell killing by the vector alone in 9L and M21, respectively. The treatment with doxorubicin alone induced 82.4% and 90.9% death in 9L and M21 cells, respectively (Figure 2). These findings prove that doxorubicin treatment is an effective strategy to improve RGD4C/AAVP-targeted gene therapy.

### 2.3. Evaluation of the Effect of Doxorubicin Treatment on Vector Cellular Trafficking

**Figure 3:**
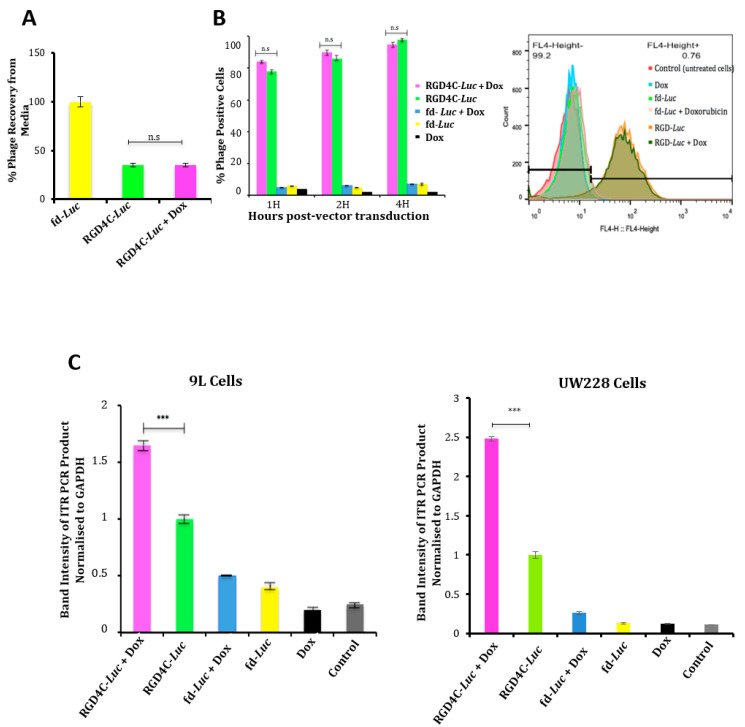
Analyses of cellular binding, internalization and nuclear accumulation of the RGD4C/AAVP vector in the presence of Dox. (**A**) Cellular attachment of the RGD4C-*Luc* vector was evaluated in 9L tumor cells in the absence or presence of Dox (0.5 μM) by quantifying the free vector in the media of 9L cells. Experiments were performed twice and in triplicate. (**B**) Vector entry in 9L cells in combination with Dox (0.5 μM). The percentage of cells stained positive for phage by FACS analysis is shown on the graph at various time points (1, 2 and 4 h) following treatment with vectors. Original FACS results after immunostaining of 9L cells are also shown. The experiments were repeated twice and carried out in triplicate. (**C**) Evaluation of the vector genome in the nucleus. 9L and UW228 tumor cells were transduced with targeted RGD4C-*Luc* or control nontargeted fd-*Luc* in the presence or absence of Dox (0.5 μΜ) or (8 μM), respectively. At day 4 post-transduction, cells were harvested and nuclei isolated. Subsequently, DNA was extracted and used as a template for PCR to quantify the amount of vector in the nuclei. Data were normalized to GAPDH (Glyceraldehyde 3-phosphate dehydrogenase). Experiments were repeated three times, and shown are the mean ± standard error of the mean (SEM) of the triplicate samples. *** *p* < 0.001 and n.s: nonsignificant.

We set out to gain understanding into the mechanism of the enhanced RGD4C/AAVP-mediated gene delivery and tumor cell killing by doxorubicin. Therefore, we investigated whether doxorubicin alters vector trafficking through the various stages of gene transfer [33,34]. First, we monitored vector attachment to the cell surface in the presence of doxorubicin by quantifying the free phage present in the medium of cells by infection of host bacteria and colony counting as previously reported [21]. Our data showed that ~37% of the phage input was recovered from the cell medium upon treatment with the RGD4C/AAVP-*Luc* vector, meaning that 63% of the added vector was attached to the tumor cell surface (Figure 3A), though the combination with doxorubicin had no effect on the attachment of the RGD4C/AAVP-*Luc* vector to the surface of cancer cells (Figure 3A). Moreover, we quantified 100% recovery of the nontargeted fd-*Luc* vector, proving no attachment to tumor cell surface. Next, internalization assays revealed that the combination of vector with doxorubicin did not increase entry of RGD4C/AAVP-*Luc* into cancer cells (Figure 3B). These data prove that doxorubicin does not affect cell attachment and entry of the RGD4C/AAVP particles into cancer cells.

**Figure 4:**
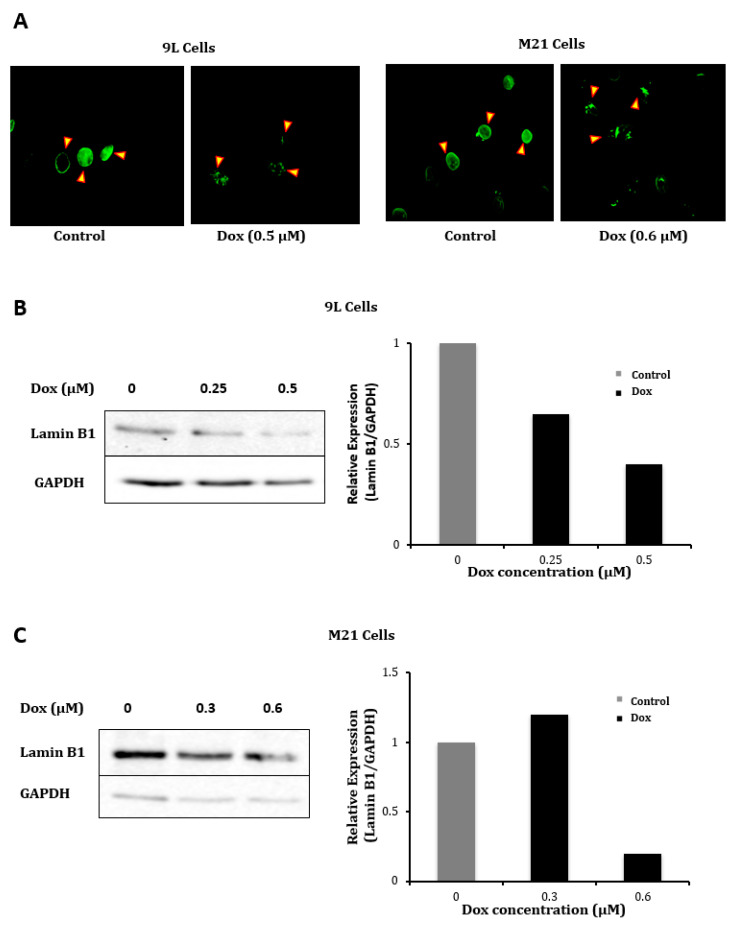
Dox reduces expression of the lamin B1 protein. (**A**) Immunofluorescence staining of the lamin B1 protein, which is a composition of the nuclear membrane, in 9L and M21 cells following Dox treatment. Pictures were taken by a fluorescent microscope. Lamin B1 staining shows that borders of the nuclear membranes (arrows) are thick in control untreated cells, while they are thinner in Dox-treated 9L and M21 cells. Original magnification ×400. (**B**,**C**) Western blot analysis of lamin B1 expression in 9L and M21 cells, respectively, following treatment with increasing doses of Dox. Graphs show quantification of the bands in the Western blots and were obtained by normalizing the lamin B1 expression to GAPDH, then presented as fold change compared to control untreated cells.

We also investigated the vector genome localization in the nucleus, since doxorubicin has been reported to delay proper chromosome condensation and nuclear envelope formation during mitosis [35], which could result in increased transport through the nuclear pores. Thus, we evaluated the nuclear accumulation of the AAVP genome as we previously reported [36] in the presence or absence of doxorubicin by quantifying the ITRs. We used these ITR cis genetic elements as targets for quantification, because they have been used to quantify the AAV genome copies (GC) in cells and because gene expression by AAVP is driven by an ITR-flanked transgene cassette or recombinant AAV genome [5,6]. Hence, nuclei were extracted from cells to perform PCR with primers reading within the AAV2 ITR domain and by using the original reported protocol [37] to semi-quantify the amount of ITRs in the nucleus. The data revealed that the combination of doxorubicin with the targeted vector resulted in a significant increase, 1.6- and 2.5-fold, of vector DNA in the nuclei of 9L and UW228 tumor cells, respectively (Figure 3C). To further validate these data, we investigated the effect of doxorubicin on expression of the lamin B1 protein, a major component of the nuclear membrane, since doxorubicin was reported to induce cell cycle arrest of the G2/M phase during which the nuclear membrane is partially produced [38,39]. Immunofluorescence staining of cells following treatment with doxorubicin showed a lower staining of lamin B1 both in 9L and M21 cells compared to untreated cells (Figure 4A). Next, Western blot analysis confirmed the drop of lamin B1 expression in 9L (Figure 4B) and M21 cells (Figure 4C) following treatment with doxorubicin in a dose-dependent manner and with the most decrease in lamin B1 obtained with the optimal doses of doxorubicin (Figure 4B,C).

### 2.4. Evaluation of Efficacy of Doxorubicin and RGD4C/AAVP Combination in a Three-Dimensional (3D) Multicellular Tumor Spheroid Model

**Figure 5:**
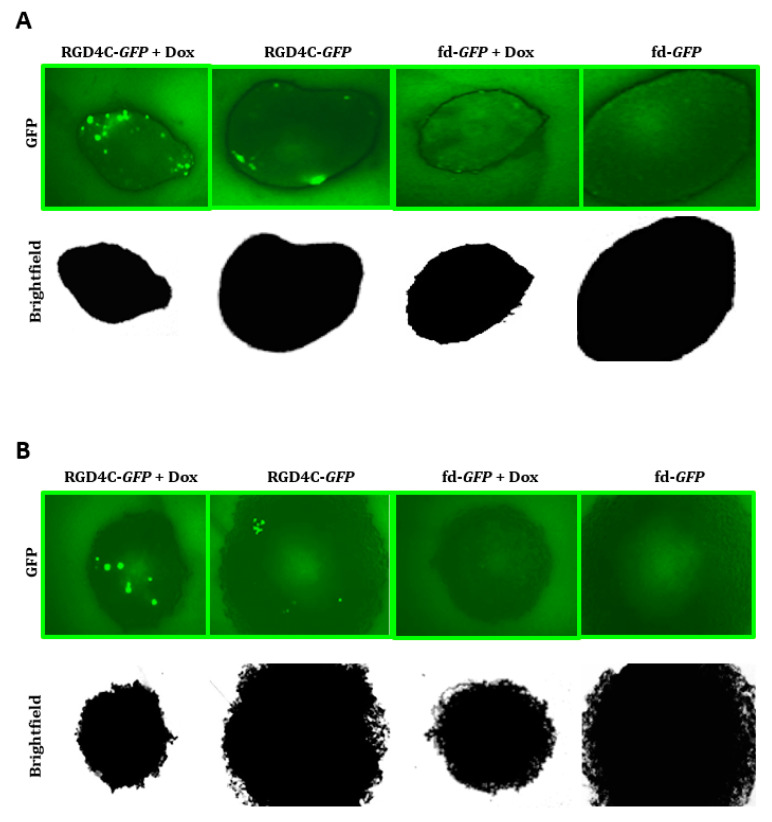
Dox increases RGD4C/AAVP transduction of 9L and M21 tumor spheres. 9L (**A**) and M21 (**B**) cells, 5 × 10^3^, were seeded into a 96-well ultra-low attachment surface plate in 200-μL complete medium. After 48 h, spheres were established in each well. The spheres were then transduced with targeted RGD4C-*GFP* or fd-*GFP* control nontargeted vectors in the presence or absence of doxorubicin (0.5 μΜ for 9L and 0.6 μΜ for M21 spheres). GFP expression was evaluated at day 10 post-transduction by using a fluorescence microscope. Original magnification ×20 for panels (**A**) and (**B**).

We sought to investigate the combination of doxorubicin and the targeted vector in 3D tumor spheres commonly used as valid models to mimic the features of tumors in vivo [21,22,36]. Therefore, we first examined gene transfer using the vector carrying the *GFP* reporter gene and monitored GFP expression over time by microscopic observation of 9L and M21 tumor spheroids (Figure 5). Only minimal GFP expression was detected in the 3D spheroids upon treatment with the targeted RGD4C/AAVP-*GFP*; however, the combination with doxorubicin significantly enhanced GFP expression from RGD4C/AAVP-*GFP* both in 9L and M21 tumor spheroids (Figure 5). The treatment with the nontargeted fd-*GFP* vector did not result in any GFP expression.

**Figure 6:**
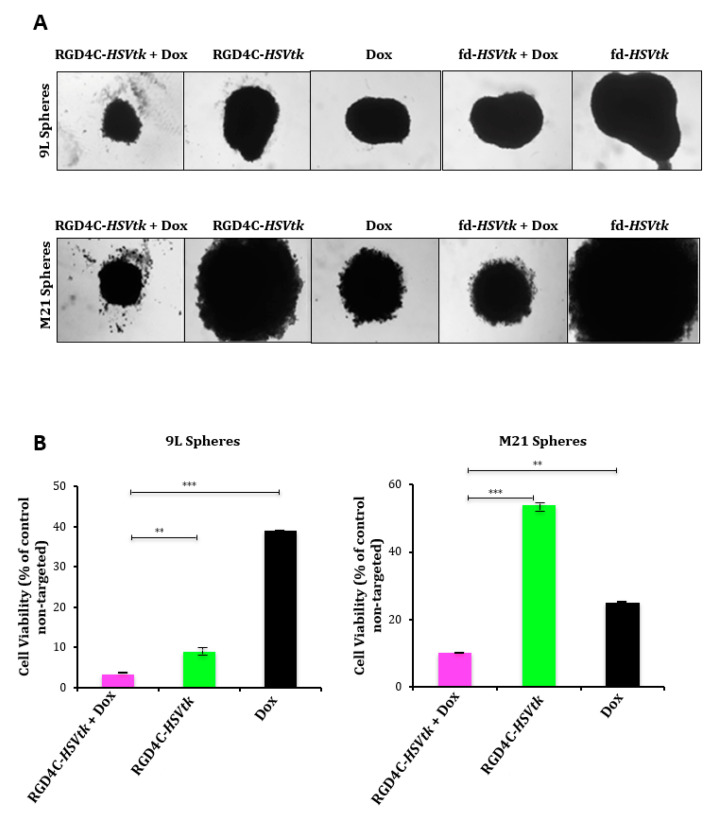
Antitumor efficacy of the combination between Dox and RGD4C/AAVP-*HSVtk* plus GCV in tumor spheres. (**A**) Bright-field images showing the size of 9L and M21 spheres following transduction with RGD4C-*HSVtk* or fd-*HSVtk*, the nontargeted vector, in the presence or absence of doxorubicin (0.5 μΜ for 9L and 0.6 μΜ for M21 spheroids). GCV was added to the spheres at day 5 post-vector transduction and renewed every 2 days. Images were taken at days 5 or 7 post-GCV treatment for 9L and M21 spheres, respectively. Original magnification ×20. (**B**) Evaluation of cell viability in 9L and M21 spheres at days 5 or 7 post-GCV treatment, respectively. Cell viability was assessed by using the Cell Titer-Glo cell viability assay. The experiments were repeated twice in triplicate, and the results shown are representative of one experiment. ** *p* < 0.01 and *** *p* < 0.001.

Next, the application of *HSVtk*/GCV suicide gene therapy on rat 9L and human M21 tumor spheroids resulted in pronounced regression of the 9L and M21 (Figure 6A) sphere volumes by the combination of doxorubicin with the targeted RGD4C/AAVP-*HSVtk* upon GCV treatment compared to individual treatments with the vector or doxorubicin alone. Subsequently, in 9L spheres, the measurement of cell viability showed that the combination of doxorubicin plus RGD4C/AAVP-*HSVtk* achieved higher tumor cell killing (~97%) than the RGD4C/AAVP-*HSVtk* vector or doxorubicin alone, which induced ~92% and ~65% cancer cell killing, respectively (Figure 6B). In M21 spheres, the measurement of cell viability showed that the combination treatment achieved higher tumor cell killing (~91%) than the RGD4C/AAVP-*HSVtk* or doxorubicin alone, which induced ~47% and ~82% cancer cell killing, respectively (Figure 6B). These findings establish that doxorubicin greatly enhances RGD4C/AAVP-mediated cancer gene therapy.

## 3. Discussion

Herein, we reported that the doxorubicin treatment of cancer cells resulted in increased RGD4C/AAVP-guided gene delivery, subsequently enhancing tumor cell killing by RGD4C/AAVP-*HSVtk* plus GCV both in 2D cell cultures and 3D tumor spheres. The combination of vector plus doxorubicin was more effective than the RGD4C/AAVP vector alone in reporter gene expression analyses than in tumor cell death experiments. However, the HSVtk/GCV strategy can produce a bystander effect, resulting in enhanced tumor cell death by the RGD4C/AAVP-*HSVtk* plus GCV [12]. Indeed, cells expressing the HSVtk can induce the death of surrounding cells via the gap junctional intercellular communications (GJIC), as previously reported [12,40]. Overtime, this “bystander effect” increases cell death by the RGD4C/AAVP-*HSVtk*, which may result in increased tumor cell death by the RGD4C/AAVP*-HSVtk* and GCV. Additionally, we used the mutant SR39 version of *HSVtk* that is more effective than the wild-type *HSVtk* [31]. Thus, the data of tumor cell death by *HSVtk* plus GCV should be partially proportional to the levels of HSVtk expression. This bystander effect could also explain the slight but insignificant effect of fd-*HSVtk* plus GCV (Supplementary Figure S1), which could be caused by unspecific phage uptake when using high vector amounts. Thus, we normalized our data to fd-*HSVtk* to subtract the effect of nonspecific uptake. Unspecific uptake of the nontargeted vector could also contribute to the slight increase of GFP-positive cells in FACS analysis experiments when treating UW228 cells with high amounts of fd-*GFP* in combination with a high doxorubicin concentration.

Nevertheless, these data are consistent with previous studies reporting that doxorubicin augments the transduction efficiency of airway cell lines by the recombinant rAAV vector [41]. Doxorubicin was also reported to increase rAAV-2 transduction in rat neuronal cell lines [30]; in that study, the authors suggested that doxorubicin improved the transduction efficiency by enhancing the nuclear translocation of rAAV. Indeed, topoisomerase II inhibitors, such as doxorubicin, have been reported to delay proper chromosome condensation and nuclear envelope formation throughout mitosis. This should increase the permeability of the nuclear membranes, subsequently facilitating nuclear transport [35]. Our data are consistent with these studies, as we found a greater vector genome in the nuclear fraction of cancer cells treated both with doxorubicin and the targeted RGD4C/AAVP vector compared to cells treated with the RGD4C/AAVP vector alone. Additionally, doxorubicin-mediated gene delivery enhancement was related to the levels of nuclear vector genome increase by this chemotherapeutic agent. Additionally, doxorubicin treatment resulted in a clear decrease of expression of the nuclear lamin B1 proteins involved in nuclear stability. During mitosis, the lamin B1 matrix is reversibly disassembled as the lamin proteins are phosphorylated [42,43,44]. Taking into account that the combination of RGD4C/AAVP with doxorubicin does not affect the vector attachment to the surface of cancer cells nor its internalization, our data postulate that doxorubicin may facilitate the nuclear translocation of the RGD4C/AAVP genome, subsequently enhancing transgene expression and consequent targeted cancer cell killing. Interestingly, we previously reported that most of drug combination treatments enhanced targeted RGD4C/AAVP gene delivery by altering the vector trafficking through obstacles to gene transfer vectors. Thus, doxorubicin action might also be mediated by its proteasome inhibitory activity [41]. Indeed, we previously reported proteasome degradation as an intracellular barrier to gene delivery by RGD4C/AAVP [24] and that proteasome-inhibiting drugs could be used to assist the phage to overcome this barrier and, subsequently, enhance gene delivery. We reported that proteasome-inhibiting drugs protect RGD4C/AAVP against proteasome degradation [24,36].

Moreover, genotoxic stress can increase AAV-mediated transduction efficiency by facilitating the conversion of single- to double-stranded DNA [45,46]. Therefore, another possible mechanism of doxorubicin-increased transduction efficiency of the RGD4C/AAVP vector could be that low-dose doxorubicin causes moderate DNA damage, resulting in activation of DNA repair enzymes, such as PARP-1(poly(ADP-ribose) polymerase-1) [29], which can replicate vector genomes. DNA repair enzymes could facilitate the conversion of the single-stranded genome of RGD4C/AAVP to double-stranded DNA, thus increasing transgene expression from the vector. This is consistent with our comet assay findings showing that the low doxorubicin doses that increased the transduction efficiency of our vector cause moderate DNA damage (Supplementary Figure S2). The comet assay is a single cell-based technique that allows the detection and quantification of DNA damage.

Together, these findings support our recent published studies reporting chemotherapy as an adjuvant to activate RGD4C/AAVP-*HSVtk* and GCV-targeted cancer gene therapy. Additionally, our current investigation has the potential to alter the clinical use of doxorubicin, and chemotherapy in general, and should influence systemic targeted cancer gene therapy by RGD4C/AAVP. Combinatorial treatment regimens represent a promising approach to overcome chemoresistance, which is a hallmark of cancer and should allow the reduction of chemotherapeutic drug administration to less toxic and cheaper doses. Moreover, both RGD4C/AAVP and doxorubicin are administered via clinical systemic routes (intravenous), suggesting that this combination treatment can be used to target distant tumors, a route that is applicable for metastatic disease. The treatment of metastatic cancer is heavily reliant upon systemically administered therapeutics. Metastases present a formidable challenge to successful cancer therapy and are responsible for as much as 90% of cancer-related deaths [47,48]. In this study, the proved efficacy of the combination between doxorubicin and RGD4C/AAVP-*HSVtk* plus GCV suicide gene therapy against M21 human melanoma provides a potential application of this chemovirotherapy against metastatic melanoma, known for its poor prognosis and less than 12 months median survival [49].

Doxorubicin is used to treat many different types of cancer and can be administered intravenously, like RGD4C/AAVP, over a course of several cycles of treatment over a few months. The dose of doxorubicin can vary according to indication and whether it is used as single therapeutic agent or in combination with other anticancer drugs. The toxicity of doxorubicin may be potentiated by other anticancer therapies and vice versa; thus, a combination might require dose adjustment [50,51]. A dose of 60–75-mg/m^2^ body surface area is recommended every three weeks when doxorubicin is used as a single agent [50,51]. When doxorubicin is administered in combination with other antitumor agents with overlapping toxicity, the dosage of doxorubicin can be reduced to 30–60 mg/m^2^ every three to four weeks [50,51]. However, bacteriophages are known for their long safety profile, since they have been administered to humans over a long time to treat bacterial infections [52] and were then approved in 2006 by the Food and Drug Administration for their use as antibacterial food additives [53]. Moreover, repeated intravenous administrations of RGD4C/AAVP carrying tumor necrosis factor alpha (TNFα), once a week over eight weeks, proved safe in dogs with natural cancers [20]. Additionally, the M13 phage displaying a peptide library, from which derived RGD4C/AAVP, was serially administered to cancer patients over a few weeks without any unwanted side effects [54]. Therefore, RGD4C/AAVP could be given safely and weekly in a combination without potential doxorubicin toxicity and might not require adjustment of the doxorubicin dosage and the interval between doxorubicin administrations.

## 4. Materials and Methods

### 4.1. Cells and Reagents

The 9L rat glioma cells were provided by Dr. Hrvoje Miletic (Univerity of Bergen, Norway). The human M21 melanoma and UW228 medulloblastoma cells were purchased from the American Type Culture Collection (ATCC, Middlesex, UK). Both cell lines were grown in Dulbecco’s modified Eagle’s medium (Sigma) with 10% fetal bovine serum (FBS, Sigma, Dorset, UK), penicillin (100 units/mL, Sigma, Dorset, UK), streptomycin (100 μg/mL, Sigma, Dorset, UK) and L-glutamine (2 mmol/L, Sigma, Dorset, UK).

### 4.2. Production, Purification and Titration of AAVP Vectors

Phage vectors were designed as previously reported [6] by inserting AAV2 transgene expression cassettes into the fUSE5 plasmid and expressing reporter or therapeutic genes under the control of the CMV promoter. Then, production and purification of AAVP phage particles were carried out as previously described [6] from the supernatant of host bacteria, followed by filtration through 0.45-μm filters. The titration of AAVP vectors was performed by colony counting following infection of the host bacteria, then expressed as bacterial transducing units (TU/μL), as previously reported [6].

### 4.3. In Vitro Cell Transduction by AAVP Vectors

The 9L and M21 cells growing in 48-well plates were incubated with targeted RGD4C/AAVP or control nontargeted AAVP vectors (1 × 10^6^ TU/cell when using *HSVtk* vectors, 2.5 × 10^5^ TU/cell for *GFP* vectors and 1 × 10^4^ TU/cell when testing *Luc* vectors) in serum-free medium. Doxorubicin was added to 9L and M21 cells at the same time with AAVP vectors. In FACS analysis experiments for GFP expression, the UW228 cells were incubated with 1 × 10^6^ TU/cell of RGD4C-*GFP* or nontargeted fd-*GFP* vectors using the same transduction protocol used for 9L and M21 cells. Following 4 h of transduction, 350 μL of complete medium containing doxorubicin were added to make up a total volume of 500 μL per well. Twenty-four hours after doxorubicin treatment, the medium was replaced with doxorubicin-free complete medium.

### 4.4. Reporter Gene Assays

Steady-Glo luciferase assay (Promega, Southampton, UK) was used to quantify luciferase expression that was then normalized to cell lysate proteins determined by the Bradford assay (Sigma, Dorset, UK). Luciferase expression was presented as relative luminescence units (RLU) per 1 μg of protein. A Nikon Eclipse TE2000-U fluorescence microscope (Tokyo, Japan) was used to monitor GFP expression.

### 4.5. Determination of Tumor Cell Killing In Vitro

Cancer cells were treated with 20-µM GCV (Sigma, Dorset, UK) at day 3 post-transduction with vectors expressing the *HSVtk* in the presence or absence of doxorubicin. Cell death was evaluated at 0, 24, 48, 72 and 96 h post-treatment with GCV by using the trypan blue exclusion methodology.

### 4.6. D Model of Multicellular Tumor Spheroid Culture and Treatment

The 9L and M21 multicellular tumor spheroids were established following 48 h culture of the tumor cells in 96-well ultra-low attachment surface plates (Corning, Nottingham, UK). Vector transduction was performed for 24 h. In combination treatment experiments, similar doxorubicin concentrations used for 2D cultures were applied for the tumor spheroids (0.5 μΜ for 9L and 0.6 μΜ for M21 spheroids). In the spheroid transduction experiments using vectors carrying the *HSVtk* gene, GCV (20 μΜ) was added to the spheroids on day 5 post-vector treatment and renewed every 2 days. The spheroid cell viability was measured by using the CellTiter-Glo assay (Promega, Southampton, UK).

### 4.7. Attachment Assay

The 9L cells were seeded in 48-well plates and grown for 48 h to reach 70–80% confluence. Cells were incubated with targeted and control nontargeted vectors carrying the *Luc* gene (1 × 10^6^ TU/cell) in serum-free medium in the presence or absence of doxorubicin (0.5 μΜ). After 1-h incubation on ice, the supernatants were harvested to quantify AAVP, as previously described [6].

### 4.8. Internalization Assay

Internalization assay on 9 L tumor cells was done as we previously reported [21]. Briefly, targeted and control nontargeted vectors carrying the *Luc* reporter gene were incubated with 9L cells at 10^4^ TU/cell at 37 °C in serum-free medium in the presence or absence of doxorubicin over a time course of 1, 2 and 4 h. To stop endocytosis, cells were cooled on ice, washed with PBS, then trypsinized to remove surface-attached phage and pelleted by centrifugation. Intracellular phage vectors were stained with a polyclonal rabbit anti-phage antibody (1:1000, Sigma, Dorset, UK) and a secondary goat anti-rabbit AlexaFluor 647 (dilution 1:500, Life Technologies, Renfrew, UK) antibody. FACS analysis was performed to measure the mean fluorescence intensity, and the results were analyzed by FlowJo software version 10.5 (TreeStar, Ashland, OR USA).

### 4.9. Nuclei Extraction

The 9L cells were first treated with targeted RGD4C/AAVP or control nontargeted vectors carrying the *Luc* reporter gene in the presence or absence of doxorubicin. At day 4 post-vector transduction, cells were harvested, and the nuclei were extracted, as previously reported [36].

### 4.10. Semi-Quantitative PCR Analysis

PCR semi-quantification of the *ITR* vector domain in the nucleus, in the presence or absence of doxorubicin, was performed as previously reported [37]. ImageJ software version 1.51j8 (NIH, Bethesda, MD USA) was used to quantify the PCR band intensity; then, data were normalized to GAPDH.

### 4.11. Comet Assay

Slides were immersed in 1% agarose in dH_2_0 at 55 °C and the agarose allowed to solidify. Cell suspension was pipetted and spread onto the first agarose layer using a coverslip. Second and third layers of melting agarose were added. Following solidification of the top layer of agarose, the slides were immersed in cold lysis buffer (2.5M-NaCl, 0.1-M Na_2_EDTA, 0.01-M Tris-HCl and 1% N- lauroylsarcosine sodium salt). The pH was adjusted to 10, followed by addition of 1% Triton X-100 and 10% DMSO before use. The slides were stored at 4 °C for at least 1 h to lyse the cells and to allow the unfolding of DNA. The slides were removed from the lysis buffer, drained and placed in a gel electrophoresis machine side-by-side. The tank was filled with fresh cold electrophoresis solution (1-mM Na_2_EDTA and 0.3-M NaOH) at 4 °C and pH 12.8. Electrophoresis was performed at 300 mA for 20 min at 4 °C in the dark to allow damaged DNA to migrate away from the nucleus, resulting in the formation of a comet “tail” whose size is proportional to the damage of DNA. Finally, at the end of electrophoresis, slides were stained with 6-diamidino-2-phenylindole (DAPI, 5 μg/mL) in mounting medium in order to view individual nuclei using a fluorescent microscope. Images of randomly selected nuclei were captured and analyzed using ImageJ software.

### 4.12. Immunofluorescence Staining for Lamin B1

Cells were grown on coverslips, then treated with doxorubicin for 24 h. Next, cells were washed with PBS and fixed in 4% paraformaldehyde for 15 min at room temperature. After fixation, cells were treated for 5 min with 50-mM ammonium chloride, followed by permeabilization with 0.2% Triton X-100 and blocking with 2% BSA in PBS (Sigma, Dorset, UK) for 1 h at room temperature. Next, cells were incubated with a primary antibody rabbit anti-lamin B1 (Abcam, Cambridge, UK); the antibody reacted both to human and rat species at 0.5 µg/mL overnight at 4 °C, followed by a secondary anti-rabbit IgG-conjugated AlexaFluor 488 (Invitrogen, Renfrew, UK) at 4.0 µg/mL for 1 h at room temperature. Finally, cells were mounted in Prolong^TM^ gold antifade mountant (Invitrogen, Renfrew, UK). Images were acquired with a Nikon Eclipse TE2000-S fluorescence microscope (Tokyo, Japan).

### 4.13. Western Blot for Lamin B1

Whole-cell lysates were prepared in radioimmunoprecipitation assay (RIPA) buffer (Sigma, Dorset, UK). After discarding the nonsoluble fraction, supernatants were subjected to SDS-PAGE and immunoblot. The membranes were incubated at 4 °C overnight under agitation with a rabbit anti-lamin B1 antibody (Abcam, Cambridge, UK) diluted 1:1000 in 5% nonfat milk in Tris-buffered saline with Tween 20. Immunoblots were performed three times, quantified by ImageJ software and normalized to GAPDH.

### 4.14. Statistical Analysis

We used GraphPad Prism software version 5.0 (GraphPad Software, Inc., San Diego, CA, USA). Data were presented as mean ± standard error of the mean (SEM). *p*-values were calculated using one-way ANOVA and Tukey tests. *p*-values are denoted as follows: * *p* < 0.05, ** *p* < 0.01 and *** *p* < 0.001; n.s: nonsignificant.

## Supplementary Materials

Figure S1: Analysis of the effects of non-targeted AAVP-*HSVtk* plus GCV and Figure S2: Comet assay: can be found at https://www.mdpi.com/1422-0067/21/21/7867/s1.
