# Supplementary material for: Doxorubicin Improves Cancer Cell Targeting by Filamentous Phage Gene Delivery Vectors
Source: Int J Mol Sci. 2020 Oct 23;21(21):7867. doi: 10.3390/ijms21217867 (PMC7660303; doi:10.3390/ijms21217867)
Supplement: Supplementary file 1 [file ijms-21-07867-s001.pdf]

Supplementary Figure 1

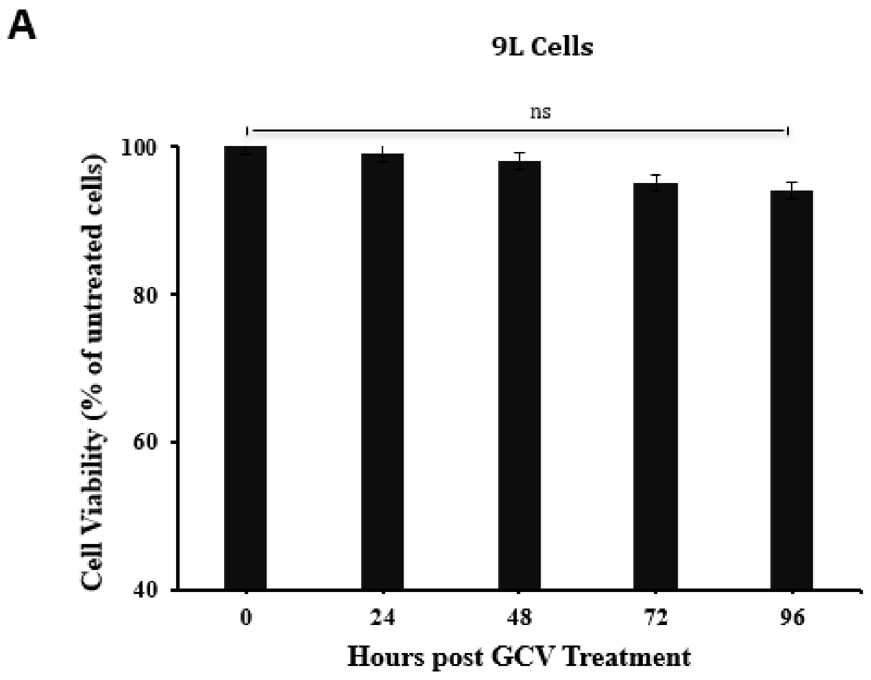

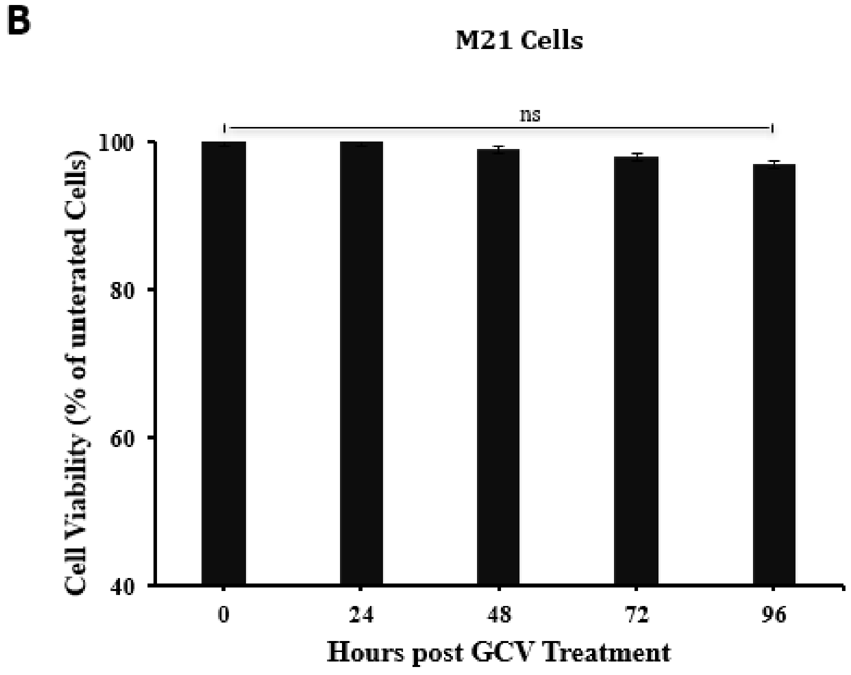

Supplementary Figure 1. Analysis of the effects of non-targeted AAVP-*HSVtk* plus GCV. 9L (**A**) and M21 (**B**) cancer cells grown in 48 well-plates, 60-80% confluent, were treated with the control non-targeted AAVP-*HSVtk* (fd-*HSVtk*) carrying the *HSVtk* gene. The cells were treated with GCV (20 μM) at day 3 post vector transduction and renewed daily. Cancer cell killing was quantified at 0, 24, 48, 72 and 96 hours post GCV treatment by using the trypan blue exclusion methodology. Results were normalized to untreated cells. The experiments were repeated twice in triplicates and the results shown are representative of one experiment. ns: nonsignificant.

Supplementary Figure 2

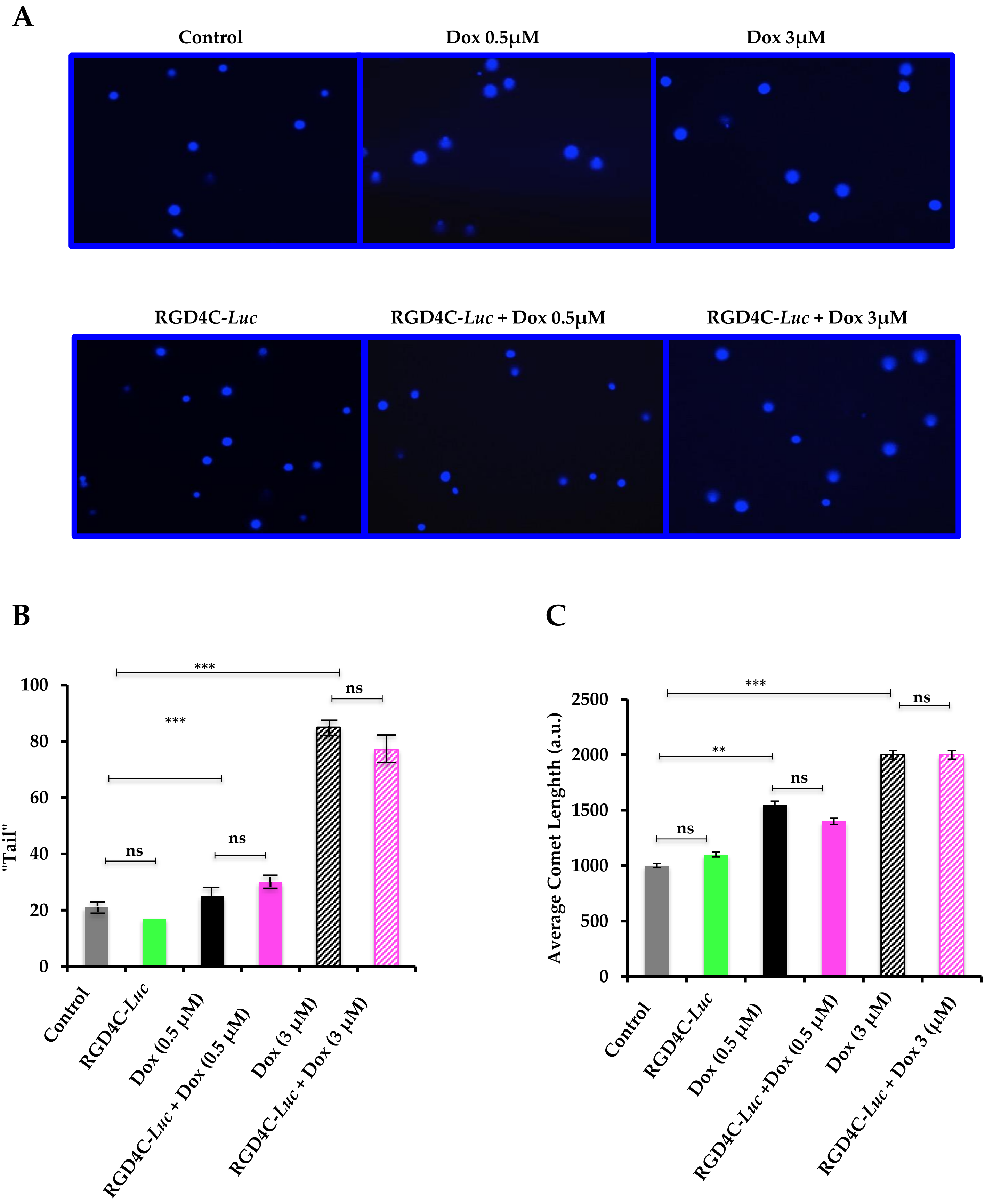

Supplementary Figure 2. Comet assay. A) Representatives images of 9L cell nuclei stained with DAPI and taken by a fluorescent microscope. **B)** Graph showing the average percentage of cell nuclei exhibiting a comet ''tail'', measured in five representative fields of view for each condition. **C)** Graph showing the average length of comets. Lengths were measured for five comets in each condition and expressed as arbitrary units (a.u.). $^{**}p < 0.01$, $^{***}p < 0.001$ and ns: nonsignificant.
